# Systematic Review of the Antitumor Activities and Mechanisms of Scorpion Venom on Human Breast Cancer Cells Lines (In Vitro Study)

**DOI:** 10.3390/jcm14093181

**Published:** 2025-05-04

**Authors:** Na-Yoen Kwon, Hyun-Kyung Sung, Jang-Kyung Park

**Affiliations:** 1 Department of Obstetrics and Gynecology, College of Korean Medicine, Ga-Chon University, Seongnam-si 13120, Republic of Korea; kwonnay@gachon.ac.kr; 2 Department of Education, College of Korean Medicine, Dongguk University, Gyeongju-si 38066, Republic of Korea; 3 Division of Clinical Medicine, School of Korean Medicine, Pusan National University, Yangsan-si 50612, Republic of Korea

**Keywords:** anticancer activity, apoptosis, breast cancer, scorpion venom

## Abstract

**Background/Objectives:** Breast cancer remains the most prevalent malignancy among women worldwide. Innovative therapies are essential to address its diverse subtypes and treatment resistance. Scorpion venom and its bioactive proteins have gained attention as potential anticancer agents owing to their multitargeted cellular effects. This review systematically evaluates their anticancer properties and mechanisms in breast cancer, highlighting therapeutic potential. **Methods:** A systematic search was conducted in five databases (PubMed, Science Direct, EMBASE, OVID, and KISS) up to September 2024. Only in vitro studies using breast cancer cell lines and investigating scorpion venom or its bioactive proteins were included. Extracted data covered study characteristics, intervention types, control groups, dose range, duration, and key outcomes. **Results:** In total, 19 studies met the eligibility criteria. Crude scorpion venom showed broad cytotoxicity against hormone receptor-positive, triple-negative, and HER2-positive breast cancer subtypes. The primary mechanisms included apoptosis induction, DNA fragmentation, oxidative stress modulation, and cell cycle regulation. Bioactive proteins, such as chlorotoxin (CTX) and Neopladine 1/2, exhibited selective anticancer effects by targeting signaling pathways, inhibiting migration and invasion, and promoting apoptosis. **Conclusion:** These findings support scorpion venom’s potential as a multitargeted anticancer agent. The complementary actions of crude venom and its proteins highlight their promise for combination therapies. Further research is needed to clarify their synergistic interactions and optimize preclinical and clinical applications.

## 1. Introduction

Breast cancer is the most prevalent cancer among women, accounting for approximately 30% of all newly diagnosed female cancers globally [1,2]. Despite advances in prevention and treatment, its incidence has steadily increased over the past 2 decades. Breast cancer remains the leading cause of cancer-related deaths globally, underscoring the urgent need for improved therapeutic approaches [3,4,5,6]. Current treatments, including mastectomy, radiation therapy, chemotherapy, and hormonal therapy, exhibit varying degrees of efficacy. Adjuvant chemotherapy effectively reduces distant recurrence risk and improves survival by targeting micrometastases [6,7]. However, chemotherapy’s adverse effects, including nausea, hair loss, early menopause, and fatigue, diminish the quality of life, emphasizing the need for alternative treatments with comparable efficacy and better safety profiles [8,9].

Natural products have gained attention as potential anticancer agents due to their favorable safety and efficacy profiles. Among them, animal venoms and toxins have attracted interest for their ability to inhibit cell growth, regulate ion channels, and induce apoptosis [8,10,11,12,13,14]. Compared to other venoms, scorpion venom exhibits unique properties, including phospholipase activity, protease inhibition, and selective cancer cell binding [15]. Scorpion crude venom refers to the unpurified extract collected directly from the venom glands of scorpions. It typically contains a heterogeneous mixture of peptides, enzymes, non-protein toxins, nucleotides, and inorganic salts. This complex composition allows crude venom to exert broad-spectrum cytotoxicity but also introduces variability in experimental outcomes. In contrast, bioactive proteins are purified peptides or proteins isolated from crude venom using biochemical techniques. These purified components exhibit more specific biological activities, often targeting defined molecular pathways. For example, purified components exhibit anticancer activities through apoptosis induction, cell-cycle arrest, oxidative stress modulation, and cell migration and invasion inhibition [16,17,18,19,20,21,22]. However, the biological significance of their mechanisms in breast cancer subtypes remains to be clarified.

Among bioactive peptides, chlorotoxin (CTX) and neopladines 1/2 have shown subtype-specific effects. CTX, originally isolated from *Leiurus quinquestriatus*, is known to inhibit cell invasion by targeting matrix metalloproteinase-2 (MMP2) and the ERα/VASP pathway. Neopladines, derived from *Tityus discrepans*, induce apoptosis via FasL signaling and have been demonstrated to have subtype-specific cytotoxicity in HER2-positive cell lines. These peptides differ in size, molecular targets, and mechanisms of action and are isolated via complex purification protocols such as high-performance liquid chromatography (HPLC), which may affect consistency across studies. Furthermore, venom composition varies significantly between scorpion species owing to differences in ecology, diet, and genetic background. Comparative studies considering species-specific venom profiles are essential for interpreting findings and identifying the most promising therapeutic candidates.

Cancer progression involves metabolic and genetic alterations, necessitating multitarget strategies [23,24,25,26,27]. Scorpion venom has therapeutic potential through its modulation of multiple pathways. Although some studies have examined its effects in breast cancer, no comprehensive, focused review exists; prior reviews have emphasized other cancer types or mechanisms in general and have often lacked depth. To address this gap, in this review, we systematically analyzed experimental studies on the use of scorpion venom in breast cancer, categorizing findings by crude venom and bioactive peptides and evaluating both their activity and mechanisms. Additionally, we underscore the clinical relevance of our findings and elucidate future research needs.

## 2. Materials and Methods

### 2.1. Data Sources and Search Strategy

Experimental studies evaluating the effects of scorpion venom on breast cancer were searched in the following electronic databases: MEDLINE (PubMed), Web of Science, EMBASE, Oriental Medicine Advanced Searching Integrated System, and Korean Studies Information Service System from inception to September 2024. Searches were conducted using a combination of terms adapted to each database: (Breast Neoplasm OR Breast Tumor OR Breast Cancer OR Mammary Cancer OR Breast Carcinoma) AND (Scorpion Venom OR Scorpion Toxin). The search covered full-text articles, titles, abstracts, and keywords. Medical Subject Headings (MeSH) terms were applied in MEDLINE to enhance search specificity. No search filters were applied to maximize inclusivity. Grey literature, such as unpublished studies or conference abstracts, was not included in the search strategy. The exclusion of grey literature may increase the potential for publication bias, which is a limitation in the interpretation of findings. This review follows the PRISMA guidelines but was not registered in PROSPERO or other databases.

### 2.2. Study Selection

The inclusion criterion was in vitro experimental studies using any breast cancer cell line. Exclusion criteria included in vivo studies, clinical trials (e.g., randomized controlled trials, case studies, case series, or case–control studies), surveys, and reviews. No restrictions were placed on the type of scorpion venom administered, allowing for diverse experimental approaches. This study had no language or publication limitations. Because this review focused on experimental studies, the use of formal risk of bias assessment tools was not applicable. However, methodological rigor was considered by evaluating experimental design consistency across studies.

### 2.3. Data Extraction

Two independent authors extracted data using a predefined data extraction form based on the Population, Intervention, Control, Outcome framework. Data collected included the first author, publication year, cancer cell model (population), test agent type (scorpion species, crude venom, or bioactive proteins) (intervention), control groups (comparison), dose range, experiment duration, minimum effective concentration, minimum effective duration, outcome measurements (outcome), and main results.

Data were categorized into crude venom and bioactive peptides, then further classified based on anticancer activity and reported mechanisms. If outcome data were insufficient, corresponding authors were contacted when possible. Disagreements in study assessments were resolved through discussion with the corresponding author. Disagreements in data extraction were resolved through discussion between the two authors; if necessary, a third reviewer was consulted to reach a consensus. Mechanisms of action were categorized based on the involved molecular pathways, including apoptosis induction, oxidative stress modulation, and cell cycle regulation.

## 3. Results

**Figure 1:**
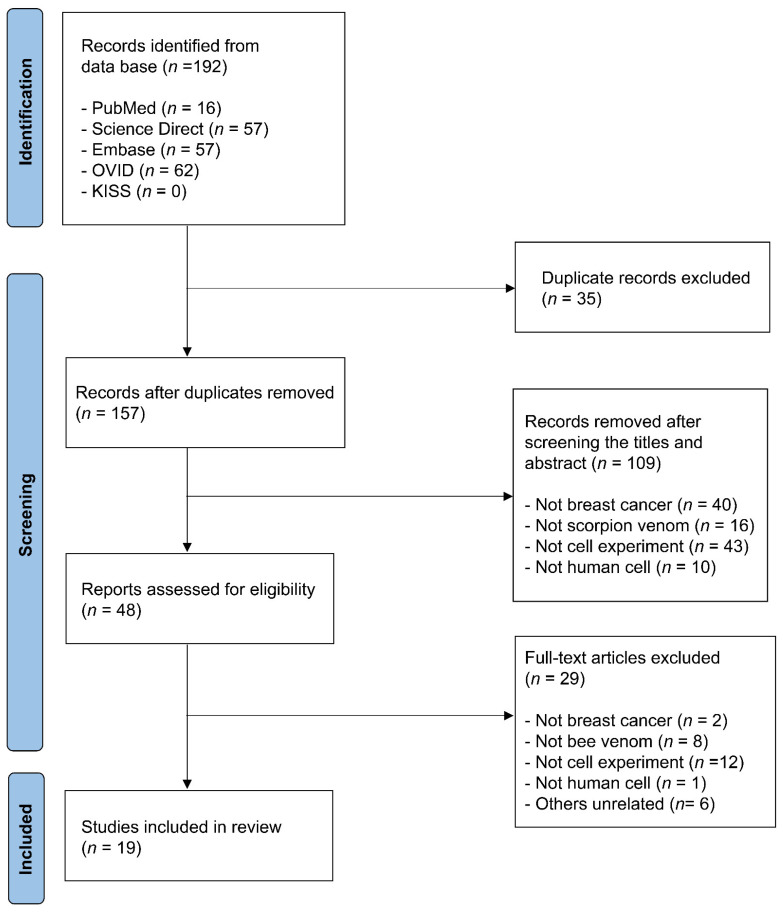
Flow chart of the review.

The initial database search yielded 192 studies. After removing 35 duplicates, the titles and abstracts of the remaining studies were screened against the inclusion criteria, leading to the exclusion of irrelevant studies. A total of 48 studies were identified for full-text review. Following a thorough assessment based on selection criteria, 19 studies were included in the final analysis. A flowchart of the study selection process is shown in Figure 1. Studies were included if they met the following criteria: (1) in vitro experimental studies, (2) use of breast cancer cell lines, and (3) evaluation of scorpion venom or bioactive proteins.

### 3.1. Analysis of Experimental Methods

Nineteen studies were reviewed to assess the anticancer effects of scorpion venom and its components in breast cancer. Among these, 15 studies utilized crude venom, while 4 focused on bioactive proteins. Various scorpion species were examined, with *Leiurus quinquestriatus* being the most frequently studied, appearing in three studies. The primary breast cancer cell lines analyzed were MCF-7, MDA-MB-231, T47D, SKBR3, and F3II. Among these, MCF-7 was the most studied, appearing in 10 studies, underscoring its role as a key experimental model for evaluating scorpion venom efficacy. Common experimental assays included MTT and XTT for cytotoxicity assessment, and Western blot and flow cytometry were frequently used to analyze apoptosis and cell-cycle regulation. However, the heterogeneity of experimental conditions—including cell-line sensitivities, assay durations, venom purification methods, and outcome measures—should be acknowledged when interpreting comparative findings across studies.

### 3.2. Analysis of Experimental Results

The 19 studies analyzed demonstrated significant growth-inhibitory effects of both crude venom and bioactive proteins on breast cancer cells. This section categorizes findings into anticancer activity and mechanisms of action, providing a detailed overview of their efficacy. For clarity, a brief summary of key findings has been provided before discussing detailed results.

#### 3.2.1. Anticancer Activity

Both crude venom and bioactive proteins exhibited significant cytotoxic effects against breast cancer cell lines, particularly MCF-7 and MDA-MB-231. These effects were largely dose- and time-dependent, with reduced cell viability observed in multiple studies.

Crude Venom: Crude venom was used in 15 studies, demonstrating broad-spectrum cytotoxicity with IC_50_ values ranging from 0.61 µg/mL to 950 µg/mL, depending on the cell line and experimental conditions. Notably, MCF-7 cells consistently exhibit sensitivity to crude venom, showing dose-dependent reductions in cell viability across multiple studies. Some studies reported significant changes in cell morphology and motility following venom exposure. Table 1 summarizes IC50 values, representing the concentration of venom required to inhibit cell viability by 50%. The outcome measures indicate key endpoints assessed in each study, such as apoptosis induction, proliferation inhibition, or oxidative stress modulation. However, variations in venom source, extraction protocol, and quantification method may affect the reliability of cross-study IC50 comparisons.

Bioactive Proteins: Proteins extracted from scorpion venom were evaluated in four studies. These proteins exhibited selective cytotoxic effects on breast cancer cell lines, with IC_50_ values of 30 µg/mL reported for leptulipin in MDA-MB-231 cells. Time- and dose-dependent effects were observed, leading to significant reductions in cell viability. These findings highlight the potential of bioactive proteins as targeted anticancer agents. The detailed results are presented in Table 2, outlining the specific proteins studied, their target mechanisms, and their effects on cell viability. Owing to the small number of protein-focused studies, broader conclusions regarding comparative efficacy remain limited and should be interpreted with caution.

#### 3.2.2. Anticancer Mechanism

The mechanisms underlying the anticancer effects of scorpion venom were categorized based on crude venom and bioactive proteins. Both forms demonstrated significant effects on key cellular processes, including apoptosis induction, cell cycle regulation, DNA fragmentation, and signaling pathway modulation.

Crude Venom: Crude venom exerts anticancer effects on breast cancer cells through multiple mechanisms. Detailed results are presented in Table 3 and include the following:

- Apoptosis induction: Crude venom upregulates pro-apoptotic genes (e.g., Bax, Caspase-3) and downregulates anti-apoptotic genes (e.g., Bcl-2). DNA fragmentation, a hallmark of apoptosis, was consistently observed in venom-treated cells.
- Cell cycle regulation: Crude venom induces cell cycle arrest at multiple phases, including G0/G1, S, and G2/M, effectively inhibiting cancer cell proliferation and disrupting mitotic progression.
- Oxidative stress modulation: Crude venom alters oxidative stress markers such as NO and GSH, contributing to increased apoptosis and cellular damage in breast cancer cells.
- Signaling protein modulation: Crude venom influences key signaling proteins involved in cancer progression, including STAT3 and IL-6, thereby disrupting pathways essential for breast cancer cell growth and survival.
- Inhibition of cell proliferation: Crude venom reduces cell viability and suppresses mitotic activity, effectively inhibiting breast cancer cell proliferation.

Proteins: Scorpion bioactive proteins have demonstrated significant anticancer effects by targeting key cellular pathways involved in breast cancer progression. These mechanisms include promoting apoptosis, inhibiting cell proliferation, migration, and invasion, and modulating the cell cycle. The detailed results of these mechanisms are summarized in Table 4.

- Apoptosis induction: Bioactive proteins such as chlorotoxin (CTX) and leptulipin enhance apoptotic processes by upregulating pro-apoptotic markers (e.g., FasL) and downregulating anti-apoptotic markers (e.g., Bcl-2). DNA fragmentation, a hallmark of apoptosis, was consistently observed in venom-treated cells, indicating effective initiation of programmed cell death. These findings highlight the ability of bioactive proteins to selectively induce apoptosis in breast cancer cells.
- Cell cycle regulation: Bioactive proteins induce cell cycle arrest at specific phases, such as G0/G1 and G2/M, by regulating cell cycle-related genes and proteins. This disruption inhibits cancer cell division and growth, emphasizing their potential to interfere with critical processes driving tumor proliferation.
- Inhibition of cell proliferation, migration, and invasion: Bioactive proteins reduce cancer cell proliferation by suppressing ERα, a key regulator in hormone-dependent breast cancer cells. Additionally, they inhibit cell migration and invasion by downregulating proteins such as MMP2 and VASP, essential for cellular motility and invasiveness. The disruption of these pathways underscores their therapeutic potential in limiting metastatic progression.

These findings suggest that scorpion venom exhibits potent cytotoxicity in breast cancer cells through multiple mechanisms. Nevertheless, caution is warranted when generalizing these findings, because the lack of in vivo validation and variability in experimental methods across studies limit their direct translational relevance. These findings support the potential of scorpion venom as a multitargeted anticancer agent and warrant further investigation in preclinical models.

## 4. Discussion

This study evaluated the anticancer effects of scorpion venom and bioactive proteins on breast cancer cells, focusing on their mechanisms of action. These findings demonstrate that both crude venom and bioactive proteins significantly inhibit breast cancer progression through multiple pathways, including apoptosis induction, inhibition of cell proliferation, and cell cycle regulation. These results highlight the potential of scorpion venom components as multitarget anticancer agents, not only for breast cancer but also for glioma, colorectal cancer, and leukemia, as supported by previous studies [32,47,48]. Previous studies have also reported similar anticancer effects of scorpion venom in glioma, colorectal cancer, and leukemia, supporting its potential as a multitargeted therapeutic agent [32,38,47,48].

The dual approach of examining both crude venom and bioactive proteins provides valuable insights into their complementary roles. Crude venom exhibited broad-spectrum effects across multiple cell lines, demonstrating dose-dependent cytotoxicity and consistent apoptosis induction. In contrast, bioactive proteins, though less extensively studied, target specific processes such as cell migration and invasion. However, the limited availability of IC_50_ data for proteins poses challenges to definitive potency comparisons with crude venom. Crude venom primarily exerts its effects through apoptosis induction via Bax/Caspase-3 activation; bioactive proteins like CTX inhibit cell migration by modulating actin cytoskeleton dynamics (Table 3 and Table 4).

Interestingly, *Leiurus quinquestriatus* and *Androctonus crassicauda* venoms effectively induced cell death in both MCF-7 and MDA-MB-231 cells, regardless of hormone receptor status. This finding is significant because these cell lines represent hormone-positive and triple-negative breast cancer subtypes, which typically require distinct therapeutic approaches such as hormone therapy and chemotherapy. The ability of these venoms to target both subtypes underscores their therapeutic versatility and potential for broad clinical applications. These findings suggest that combining scorpion venom components with existing therapies could enhance treatment efficacy, particularly in cases where conventional treatments show a limited response. Further research exploring combination therapies with targeted agents or chemotherapy could provide valuable insights into the optimization of the clinical application of scorpion venom components.

These findings align with previous studies investigating the anticancer potential of various scorpion venoms and their derived proteins. Nosouhian et al. (2024) [49] demonstrated that crude *Hottentotta saulcyi* venom effectively inhibits breast cancer cell proliferation in both in vitro and in vivo models, further supporting the broad-spectrum cytotoxic effects observed in our study. Additionally, Khalid et al. (2024) [50] proposed a computationally designed fusion protein, Leptulipin-p28, with multitarget anticancer activity against breast cancer. These findings emphasize the potential of bioactive proteins as targeted therapeutics and complement our observation that venom proteins exert distinct regulatory effects on cancer cell behavior. Furthermore, Seifi et al. (2023) [51] developed a chimeric peptide (MeICT/IMe-AGAP) derived from *Mesobuthus eupeus* venom, demonstrating the feasibility of engineering venom-based therapeutic molecules with enhanced efficacy.

Over the past decade, research has increasingly focused on the apoptotic and antiproliferative effects of scorpion venoms. Despite this progress, challenges remain in translating these findings into clinically viable treatments, particularly concerning bioavailability, toxicity, and mechanisms of action in complex biological environments.

Further studies are needed to evaluate bioactive proteins in more comprehensive experimental settings and quantify their therapeutic potential. Additionally, exploring the synergy between scorpion venom components and existing breast cancer therapies may pave the way for novel multitarget therapeutic strategies.

### 4.1. Comparative Mechanistic Insights into Crude Venom and Bioactive Protein

This study highlights the complementary yet distinct anticancer mechanisms of crude venom and bioactive proteins, including CTX from *Leiurus quinquestriatus* and neopladine 1/2 from *Tityus discrepans*.

Crude venom exhibits broad-spectrum cytotoxicity across various breast cancer cell lines, primarily through the upregulation of pro-apoptotic markers (e.g., Bax, Caspase-3) and the downregulation of anti-apoptotic markers (e.g., Bcl-2). Its generalized cytotoxicity is attributed to a complex mixture of bioactive compounds that affect multiple cellular pathways.

In contrast, bioactive proteins act through selective mechanisms targeting specific pathways. CTX inhibits the ERα/VASP signaling pathway in hormone receptor-positive breast cancer cells (MCF-7) and reduces MMP2 expression, limiting cell migration and invasion. Additionally, CTX exerts significant anticancer effects in triple-negative breast cancer (MDA-MB-231) via alternative mechanisms.

Neopladine 1/2 from *Tityus discrepans* primarily induces apoptosis in HER2-positive cells (SKBR3) through the extrinsic apoptotic pathway, upregulating FasL and downregulating Bcl-2. Interestingly, the combined application of neopladines 1 and 2 reduced apoptosis but increased necrosis, suggesting complex interactions requiring further investigation. This shift from apoptosis to necrosis may indicate a dose-dependent cytotoxic response, where higher concentrations of these proteins disrupt controlled cell death pathways and lead to secondary necrosis. While apoptosis is a regulated form of cell death that minimizes inflammatory responses, necrosis can trigger immune activation and alter the tumor microenvironment. Understanding whether this transition enhances therapeutic efficacy or introduces unintended inflammatory risks will be crucial for potential clinical applications.

This comparison suggests that while crude venom exerts broad and potent cytotoxic effects, bioactive proteins like CTX and neopladine provide precision by targeting specific molecular pathways. Combining these approaches could facilitate the development of multitarget therapeutic strategies tailored to different breast cancer subtypes.

Taken together, scorpion venom and its components exert anticancer effects through specific molecular pathways. Crude venom typically activates the intrinsic apoptotic cascade via Bax and Caspase-3, while suppressing anti-apoptotic factors like Bcl-2. Furthermore, it modulates pro-tumorigenic signaling by downregulating IL-6 and STAT3. In contrast, bioactive proteins demonstrate more selective activity. CTX interferes with the ERα/VASP and MMP2 pathways to suppress migration and invasion in hormone receptor-positive cells, while neopladines 1 and 2 trigger extrinsic apoptosis in HER2-positive cells through FasL upregulation. These signaling interactions highlight the molecular basis for their multitargeted anticancer actions.

### 4.2. Mechanistic Insights Based on Target Cells

Scorpion venoms exhibit distinct anticancer mechanisms depending on breast cancer subtypes, including hormone receptor-positive, triple-negative, and HER2-positive breast cancer cells. This section provides a detailed analysis of interactions between different scorpion venoms and specific breast cancer cell types.

#### 4.2.1. Hormone Receptor-Positive Breast Cancer Cells (MCF-7, T47D)

In hormone receptor-positive breast cancer cells, such as MCF-7 and T47D, venoms from *Leiurus quinquestriatus*, *Buthus martensii Karsch*, *Hottentotta schach*, *Androctonus crassicauda*, and *Odontobuthus doriae* exhibited significant anticancer effects. These venoms consistently upregulated pro-apoptotic genes such as Bax and Caspase-3 while downregulating anti-apoptotic markers like Bcl-2, thereby promoting apoptosis. DNA fragmentation, a hallmark of apoptosis, was observed in cells treated with *Androctonus amoreuxi, Androctonus crassicauda*, and *Odontobuthus doriae,* reinforcing their roles in programmed cell death.

Increased oxidative stress is another key mechanism, with *Hottentotta schach, Androctonus crassicauda*, and *Odontobuthus doriae* venoms elevating reactive oxygen species (ROS) and nitric oxide (NO) while reducing glutathione (GSH) levels, leading to amplified cellular damage and apoptosis. These multifaceted mechanisms highlight the therapeutic potential of scorpion venom for targeting hormone receptor-positive breast cancer cells. Given the reliance on hormone therapy for these cancers, scorpion venom components could serve as complementary agents to enhance apoptosis in cases with developing resistance to endocrine therapy. Exploring their synergy with existing selective estrogen receptor modulators (SERMs) or aromatase inhibitors could offer new therapeutic strategies.

#### 4.2.2. Triple-Negative Breast Cancer Cells (MDA-MB-231, F3II)

Triple-negative breast cancer cells, including MDA-MB-231 and F3II, were sensitive to venoms from *Rhopalurus junceus*, *Androctonus bicolor*, *Androctonus crassicauda*, and *Leiurus quinquestriatus.* These venoms modulated gene expression by upregulating pro-apoptotic markers such as Bax and Caspase-3 while downregulating anti-apoptotic markers like Bcl-2, strongly promoting apoptosis in these highly aggressive and treatment-resistant breast cancer cells.

Moreover, *Androctonus bicolor, Androctonus crassicauda*, and *Leiurus quinquestriatus* venom induced cell cycle arrest at the G0/G1 and G2/M phases, significantly inhibiting proliferation. DNA fragmentation was also observed, reinforcing its role in apoptosis induction. Additionally, these venoms modulated cytokine expression, altering the tumor microenvironment and reducing conditions favorable for cancer progression. Given that targeted therapies are lacking for triple-negative breast cancer and chemotherapy is often employed, these bioactive agents could offer novel approaches to overcoming chemoresistance by modifying key regulatory pathways involved in tumor progression.

#### 4.2.3. HER2-Positive Breast Cancer Cells (SKBR3)

In HER-2 positive breast cancer cells (SKBR3), *Tityus discrepans* venom demonstrated significant efficacy in modulating apoptosis-related markers, upregulating pro-apoptotic proteins such as Fas ligand (FasL), and downregulating anti-apoptotic markers like Bcl-2, effectively inducing apoptosis in SKBR3 cells.

Additionally, bioactive proteins, particularly neopladines 1 and 2 from *Tityus discrepans*, enhanced the pro-apoptotic effects of crude venom. These proteins further increased the expression of apoptosis-inducing markers while suppressing anti-apoptotic factors, suggesting a more targeted and potentially safer therapeutic option. This specificity underscores the potential of bioactive proteins in precision medicine for HER2-positive breast cancer treatment. Considering the established use of HER2-targeted therapies such as trastuzumab, exploring whether bioactive proteins can enhance the efficacy of these treatments or overcome resistance mechanisms is a potentially promising area of future research.

### 4.3. Limitations of the Study

This review provides valuable insights into the anticancer effects of scorpion venom and bioactive proteins on breast cancer cells; however, several limitations must be acknowledged. One key limitation is the imbalance in the number of studies investigating crude venom (*n* = 15) compared to those investigating bioactive proteins (*n* = 4), which may limit the interpretability and generalizability of comparative findings. This disparity makes it difficult to determine whether observed differences in therapeutic effects are due to actual biological variations or a research bias favoring crude venom studies. Addressing this imbalance in future studies will be critical for drawing more definitive conclusions about their relative efficacy. Furthermore, the lack of in vivo validation prevents assessment of pharmacokinetics, toxicity, and immune interactions, which are critical for clinical translation.

Additionally, many protein-based studies lack critical quantitative data, such as IC_50_ values or detailed dose–response relationships, making potency assessments difficult. The absence of standardized experimental protocols and inconsistent reporting of cytotoxic concentrations hinder direct comparisons between studies. Owing to substantial heterogeneity across studies—including differences in cell models, venom preparation methods, and outcome measures—conducting a meta-analysis was not feasible and was consequently not attempted in this review. This limitation affects the ability to determine optimal dosing strategies and therapeutic windows for bioactive proteins. Future research should focus on generating comprehensive pharmacokinetic and pharmacodynamic profiles to facilitate clinical translation.

Another limitation is the absence of studies directly investigating the synergistic effects of crude venom and bioactive proteins. Although complementary mechanisms have been observed, combination treatment experiments have not been conducted, leaving unanswered questions regarding their potential for enhanced therapeutic efficacy.

Furthermore, despite the diversity of breast cancer subtypes studied, the limited range of cell lines and experimental conditions reduces the generalizability of findings. Expanding studies to include drug-resistant and metastatic breast cancer subtypes may strengthen clinical relevance. Notably, scorpion venom collection poses ethical and practical challenges, including concerns related to animal welfare, large-scale harvesting, and quality standardization. These challenges highlight the importance of investigating recombinant or synthetic peptide analogs as feasible alternatives for future therapeutic development.

Moreover, although this review included studies involving multiple scorpion species, comparative conclusions on their relative efficacy could not be drawn. This limitation is primarily due to the lack of standardized conditions across studies, such as differences in venom extraction methods, peptide composition, and target assays, which prevent direct interspecies comparisons.

These limitations highlight the need for more balanced and comprehensive studies to fully evaluate the therapeutic potential of scorpion venom and bioactive proteins in breast cancer treatment. Future studies should incorporate robust experimental designs with standardized methodologies, ensuring reproducibility and facilitating direct comparisons across different bioactive compounds.

### 4.4. Future Research Directions

To expand current findings, future research should prioritize broadening in vitro studies to include a wider range of bioactive proteins, allowing for a more comprehensive comparison with crude venom. These studies should also incorporate detailed dose–response experiments and IC_50_ measurements to enable direct comparisons. Standardizing experimental protocols will facilitate a more reliable evaluation of bioactive proteins’ anticancer efficacy and support their integration into preclinical and clinical trial designs. Furthermore, studies assessing long-term effects of repeated venom exposure on normal cells and tissues will be critical in determining potential toxicity risks.

Additionally, investigating the synergistic potential of crude venom and purified bioactive proteins may contribute to the development of more effective multitarget therapeutic strategies. Optimizing dosing regimens and delivery methods, such as nanoparticle-based delivery systems or targeted drug conjugates, could enhance therapeutic efficacy while minimizing toxicity and off-target effects. These advancements will help determine whether bioactive compounds can be integrated into existing treatment regimens, either as adjuvant therapies or standalone agents.

While most of the current findings are derived from in vitro models, future research should emphasize the translation of these results into in vivo systems. In particular, animal studies using xenograft or syngeneic breast cancer models are essential to evaluate the pharmacokinetics, biodistribution, and systemic toxicity of venom-derived compounds. Although in vivo studies on scorpion venom in breast cancer are limited, prior research in other cancer models, including glioma and colorectal cancer, has demonstrated systematic antitumor efficacy, providing preliminary support for translational potential. Integrating these findings with immune profiling and tumor microenvironment dynamics will potentially offer critical insights for clinical applicability.

Expanding in vitro studies to include drug-resistant, metastatic, and underrepresented breast cancer subtypes will improve the applicability of these findings. This could involve testing venom components in co-culture models that better replicate the tumor microenvironment, allowing for a more precise assessment of their interactions with cancer cells. Addressing these areas will refine our understanding of scorpion venom’s therapeutic potential, advancing translational applications while remaining within the preclinical research scope.

Ultimately, the integration of bioactive compounds into clinical research pipelines will require a multidisciplinary approach involving molecular oncology, pharmacology, and clinical trial development to ensure their safe and effective use in human patients. Future research should also explore the combination of bioactive compounds with standard cancer therapies to assess potential benefits in overcoming drug resistance and enhancing treatment efficacy.

## 5. Conclusions

This study highlights the significant anticancer potential of crude scorpion venom and bioactive proteins against various breast cancer subtypes. Crude venom exhibits broad-spectrum cytotoxic effects by inducing apoptosis, DNA fragmentation, and oxidative stress, contributing to its potent anticancer activity. In contrast, bioactive proteins such as CTX and neopladines 1 and 2 exert more targeted effects by selectively modulating apoptosis-related signaling pathways.

These findings suggest that integrating the broad cytotoxicity of crude venom with the targeted precision of bioactive proteins could enable the development of effective multitarget therapeutic strategies for breast cancer treatment. The potential for synergy between these components warrants further investigation to determine whether their combined application can enhance therapeutic efficacy while minimizing toxicity. Additionally, research should focus on optimizing delivery methods, establishing standardized dosing regimens, and evaluating interactions with existing breast cancer treatments.

Further preclinical and translational studies are needed to bridge the gap between laboratory findings and clinical applications. Expanding research into drug-resistant and metastatic breast cancer models and conducting in vivo studies will be crucial in validating the therapeutic potential of scorpion venom components.

## Figures and Tables

**Table 1:** Anticancer activity of crude scorpion venom in breast cancer cells.

Study ID	Author (Year)	Target Cells	Scorpion Species	Concentration	IC_50_	Duration	Outcome Measure	Results
1	D’Suze et al. (2010) [28]	SKBR3	*Tityus discrepans*	1, 5, 15, 30 µg/µL	n.r	1, 2, 3, 4, 5, 6 h	1. Cytotoxicity (MMT)	1. Dose- and time-dependent increase
2	Erdeş et al. (2014) [29]	MCF-7	*Leiurus abdullahbayrami*	200 µg/mL	n.r	24, 48 h	1. Cytotoxicity (XTT)	1. No effect
3	Salama et al. (2021) [30]	MCF-7	*Leiurus quinquestriatus*	0–50 µg/mL	8.86 ± 0.7 µg/mL	24 h	1. Cytotoxicity (MMT)	1. Dose-dependent increase
4	Said et al. (2022) [31]	MCF-7	*Leiurus quinquestriatus*	1–1000 µg/mL	100 µg/mL	24 h	1. Cytotoxicity (MMT)	1. Dose-dependent increase
				100 µg/mL			1. Cell morphology	1. Changed
5	Al-Asmari et al. (2016) [32]	MDA-MB-231	*Androctonus crassicauda* (V1)	50, 100 µg/mL	n.r	24 h	1. Cell motility 2. Colony formation	1. Decrease (50 ^a^/100 ^a^) 2. Decrease (100 ^a^)
			*Androctonus bicolor* (V2)	50, 100 µg/mL	n.r	24 h	1. Cell motility 2. Colony formation	1. Decrease (50 ^a^/100 ^a^) 2. Decrease (100 ^a^)
			*Leiurus quinquestriatus* (V3)	50, 100 µg/mL	n.r	24 h	1. Cell motility 2. Colony formation	1. Decrease (50 ^a^/100 ^a^) 2. Decrease (50 ^a^/100 ^a^)
6	Salem et al. (2016) [33]	MCF-7	*Androctonus amoreuxi*	0.01, 0.1, 1, 10, 100 µg/mL	0.61 µg/mL	24, 48, 72 h	1. Cytotoxicity (SRB)	1. Increase
7	Li et al. (2014) [34]	MCF-7	*Buthus martensii Karsch*	100, 200, 400, 600, 800 µg/mL	n.r	4, 12, 16, 24 h	1. Cytotoxicity	1. Dose- and time-dependent increase
8	Dezianian et al. (2020) [35]	MCF-7	*Hottentotta schach*	25, 50, 100, 200 µg/mL	n.r	24 h	1. Cytotoxicity (1) MTT (2) NRU	1. (1) Increase (25 ^a^/50 ^c^/100 ^d^/200 ^d^) (2) Inhibited (25 ^a^/50 ^e^/100 ^e^/200 ^e^)
				100, 200 µg/mL			1. Cell morphology	1. Changed
9	Díaz-García et al. (2019) [36]	F3II cells	*Rhopalurus junceus*	0.1, 0.5, 0.75, 1 mg/mL	0.95 ± 0.17 mg/mL	72 h	1. Cytotoxicity (MTT)	1. Increase (0.5 ^a^/0.75 ^b^/1 ^b^)
10	Díaz-García et al. (2017) [37]	MDA-MB-231	*Rhopalurus junceus*	0.12, 0.25, 0.5, 0.75, 1 mg/mL	0.75 mg/mL	72 h	1. Cytotoxicity (MTT)	1. Increase (0.5 ^a^/0.75 ^b^/1 ^b^)
11	Al-Asmari et al. (2016) [38]	MDA-MB-231	*Androctonus bicolor*	100–1000 µg/mL	839 ± 8 µg/mL (24 h), 753 ± 6 µg/mL (48 h)	24, 48 h	1. Cytotoxicity (MTT)	1. Dose- and time-dependent increase
				839 ± 8, 753 ± 6 µg/mL		24, 48 h	1. Cell morphology	1. Changed ^a^
12	Zargan et al. (2011) (A) [39]	MCF-7	*Androctonus crassicauda*	10, 25, 50, 100, 200 μg/mL	269 μg/mL	24 h	1. Cytotoxicity (1) MMT assay (2) LDH assay	1. (1) Dose-dependent increase (20 ^a^/200 ^c^) (2) Significant release (50 ^b^/100 ^c^)
				50, 100 μg/mL			1. Cell morphology	1. Changed in a dose-dependent manner
13	Zargan et al. (2011) (B) [40]	MCF-7	*Odontobuthus doriae*	10, 25, 50, 100, 200 μg/mL	n.r	24 h	1. Cytotoxicity (1) MMT assay (2) LDH assay	1. (1) Dose-dependent increase (10 ^b^/20 ^c^/50 ^c^/100 ^c^/200 ^c^) (2) Significant release (10 ^a^/100 ^c^)
				50, 100 μg/mL			1. Cell morphology	1. Changed (50/100)
14	Al-Asmari et al. (2018) [41]	MDA-MB-231	*Androctonus crassicauda* (V1)	500–1000 μg/mL	950 μg/mL 900 μg/mL	24 h	1. Cytotoxicity (MTT)	1. Dose-dependent increase
			*Leiurus quinquestriatus* (V2)					
15	Al-Asmari et al. [42] (2017)	MDA-MB-231	*Androctonus crassicauda* (V1)	80 μg/mL	n.r	24, 48 h	1. DNA damage	1. Induced
			*Androctonus bicolor* (V2)					
			*Leiurus quinquestriatus* (V3)					

^a^ *p* < 0.05; ^b^ *p* < 0.01; ^c^ *p* < 0.005; ^d^ *p* < 0.001; ^e^ *p* < 0.0001. n.r, not reported; MMT, Methylthiazolyldiphenyl-tetrazolium bromide assay; XTT, 2,3-Bis(2-Methoxy-4-Nitro-5-Sulfophenyl)-5-[(Phenylamino)Carbonyl]-2H-Tetrazolium Hydroxide assay; SRB, Sulforhodamine B assay; MTT, 3-(4,5-Dimethylthiazol-2-yl)-2,5-Diphenyltetrazolium Bromide assay; NRU, Neutral Red Uptake assay; LDH, Lactate Dehydrogenase release assay.

**Table 2:** Anticancer activity of proteins extracted from scorpion venom in breast cancer cells.

Study ID	Author (Year)	Target Cells	Scorpion Species	Peptide	Concentration	IC_50_	Duration	Outcome Measure	Results
16	Pedron et al. (2018) [43]	MCF-7	*Vaejovis mexicanus* *smithi*	VmCT1	0.09–50 µmol/L	n.r	4, 24 h	1. Cell viability	1. Reduction (25 ^b^/50 ^a^ at 4 h)
1	D’Suze et al. (2010) [28]	SKBR3	*Tityus discrepans*	Neopladine 1,2	1, 5, 15, 30 µg/µL	n.r	1, 2, 3, 4, 5, 6 h	1. Cell morphology	1. Changed
2	Erdeş et al. (2014) [29]	MCF-7	*Leiurus abdullahbayrami*	Etoposide	60 µM	n.r	24, 48 h	1. Cell viability	1. Significant reduction (4 h ^b^/48 h ^c^)
17	Rezaei et al. (2022) [44]	MDA-MB-231	*Hemiscorpius Lepturus*	Purified Leptulipin	6.25, 12.5, 25, 50 µg/mL	30 µg/mL	24 h	1. Cytotoxicity (1) MTT (2) LDH 2. Cell morphology	1. (1) Increase (12.5 ^b^/25 ^c^/50 ^c^) (2) Increase (25 ^c^/50 ^d^) 2. Changed

^a^ *p* < 0.05; ^b^ *p* < 0.01; ^c^ *p* < 0.005; ^d^ *p* < 0.001. n.r, not reported; MTT, 3-(4,5-Dimethylthiazol-2-yl)-2,5-Diphenyltetrazolium Bromide assay; LDH, Lactate Dehydrogenase release assay.

**Table 3:** Anticancer mechanism of crude scorpion venom in breast cancer cells.

Study ID	Author, Year	Target Cells	Scorpion Species	Concentration	IC_50_	Duration	Outcome Measure	Results
1	D’Suze et al. (2010) [28]	SKBR3	*Tityus discrepans*	1, 5, 15, 30 µg/µL	n.r	1, 2, 3, 4, 5, 6 h	1. Pro-apoptotic protein 2. Anti-apoptotic gene	1. FasL: Upregulated (n.r) 2. Bcl-2: Downregulated (n.r)
4	Said et al. (2022) [31]	MCF-7	*Leiurus quinquestriatus*	100 µg/mL	100 µg/mL	24 h	1. Apoptosis 2. Cell cycle: S phase 3. Gene expression (1) Pro-apoptotic genes (2) Anti-apoptotic genes	1. Significant elevation ^a^ 2. Significant elevation ^a^ 3. (1) Bax, Caspase-3, and Caspase-9: Upregulated ^a^ (2) Bcl-2, ALDOA, PKM: Downregulated ^a^
6	Salem et al. (2016) [33]	MCF-7	*Androctonus amoreuxi*	0.5 µg/mL	0.61 µg/mL	24 h	1. DNA fragmentation	1. Induced
7	Li et al. (2014) [34]	MCF-7	*Buthus martensii Karsch*	600 µg/mL	n.r	24 h	1. Gene expression (1) Pro-apoptotic genes (2) Anti-apoptotic genes 2. Cell cycle (1) G0/G1 phase (2) G2/M phase (3) S phase 4) Cell cycle related protein	1. (1) Caspase-3: Upregulated (n.r) (2) Bcl-2: Downregulated (n.r) 2. (1) Significant elevation^a^ (2) No significant change (3) Significant reduction (4) Cyclin D1: Decrease (n.r)
8	Dezianian et al. (2020) [35]	MCF-7	*Hottentotta schach*	25, 50, 100, 200 µg/mL	n.r	24 h	1. Oxidative stress (1) NO (2) Catalase enzyme activity (3) GSH content 2. Apoptosis 3. Pro-apoptotic gene	1. (1) Increase (25 ^a^/50 ^c^/100 ^d^/200 ^d^) (2) Decrease (25 ^c^/50 ^d^/100 ^d^/200 ^d^) (3) Decrease (25 ^d^/50 ^d^/100 ^d^/200 ^d^) 2. Significant elevation (50 ^d^/100 ^d^/200 ^d^) 3. Capase-3: Upregulated (25 ^c^/50 ^d^/100 ^d^/200 ^d^)
9	Díaz-García et al. (2019) [36]	F3II cells	*Rhopalurus junceus*	0.5 mg/mL	0.95 ± 0.17 mg/mL	24, 48 h	1. Apoptosis 2. Gene expression (1) Pro-apoptotic genes (2) Anti-apoptotic genes	1. Significant elevation: Early stage ^b^, late stage ^a^ 2. (1) Upregulated: p53 (24 h ^b^/48 h ^c^), bax (48 h ^c^), Caspase-3 (48 h ^c^) (2) Downregulated: bcl-2 (24 h ^a^/48 h ^c^)
10	Díaz-García et al. (2017) [37]	MDA-MB-231	*Rhopalurus junceus*	0.375 mg/mL	0.75 mg/mL	48 h	1. Apoptosis	1. Significant elevation ^b^
				0.375 mg/mL		24, 48 h	1. Gene expression (1) Pro-apoptotic genes (2) Anti-apoptotic genes	1. (1) Upregulated: p53 (24 h ^a^/48 h ^c^), Bax (24 h ^b^/48 h ^b^), Puma (24 h ^b^/48 h ^c^), Noxa (24 h ^b^/48 h ^c^), Caspase-3 (24 h ^a^/48 h ^a^), p21 (24 h ^a^/48 h ^a^) (2) Downregulated ^a^: Bcl-2 (48 h ^c^), Bcl-xL (24 h ^a^/48 h ^c^)
11	Al-Asmari et al. (2016) [38]	MDA-MB-231	*Androctonus bicolor*	839 ± 8, 753 ± 6 µg/mL	839 ± 8 µg/mL (24 h), 753 ± 6 µg/mL (48 h)	24, 48 h	1. Cell cycle: G0/G1 phase	1. Significant elevation (24 h ^a^/48 h ^a^)
11	Zargan et al. (2011) (A) [39]	MCF-7	*Androctonus crassicauda*	50, 100 μg/mL	269 μg/mL	24 h	1. MMP 2. Oxidative stress: NO 3. Gene expression: Caspase-3 4. DNA fragmentation 5. Cell proliferation	1. Significant increase (100 ^b^) 2. Significant increase (100 ^b^) 3. Significant increase (50 ^b^, 100 ^b^) 4. Induced (50) 5. Inhibited (50 ^b^/100 ^c^)
13	Zargan et al. (2011) (B) [40]	MCF-7	*Odontobuthus doriae*	50, 100 μg/mL	n.r	24 h	1. MMP 2. Oxidative stress (1) NO (2) GSH 3. Gene expression: Caspase-3 4. DNA fragmentation 5. Cell proliferation	1. Significant increase (50 ^a^/100 ^c^) 2. (1) Significant increase (50 ^b^/100 ^c^) (2) Significant decrease (50 ^c^/100 ^c^) 3. Significant increases (50 ^a^/100 ^c^) 4. Induced (50/100) 5. Inhibited (50 ^a^/100 ^c^)
14	Al-Asmari et al. (2018) [41]	MDA-MB-231	*Androctonus crassicauda* (V1)	950 μg/mL	950 μg/mL	24, 48 h	1. ROS 2. Cell cycle: G2/M phase 3. Morphological assessment	1. Significant increase (24 h ^a^) 2. Significant elevation (24 h ^a^/48 h ^a^) 3. Significant change (24 h ^a^/48 h ^a^)
			*Leiurus quinquestriatus* (V2)	900 μg/mL	900 μg/mL			
15	Al-Asmari et al. (2017) [42]	MDA-MB-231	*Androctonus crassicauda* (V1)	80 μg/mL	n.r	24, 48 h	1. DNA damage	1. Induced
						22 h	1. Cell invasion	1. Inhibited ^a^
						12 h	1. Gene expression (1) Pro-apoptotic genes (2) Anti-apoptotic genes 2. Signaling protein 3. Cytokine	1. (1) p53: Upregulated^a^ (2) Bcl-xL, BID: Downregulated ^a^ 2. STAT3, RhoC: Inhibited ^a^, Erk^1/2^: n.s 3. IL-6: Decreased^a^
			*Androctonus bicolor* (V2)			24, 48 h	1. DNA damage	1. Induced
						22 h	1. Cell invasion	1. Inhibited^a^
						12 h	1. Gene expression (1) Pro-apoptotic genes (2) Anti-apoptotic genes 2. Signaling protein 3. Cytokine	1. (1) p53: Upregulated ^a^ (2) Bcl-xL, BID: Downregulated ^a^ 2. STAT3, RhoC, Erk^1/2^: Inhibited ^a^ 3. IL-6: Decreased ^a^
			*Leiurus quinquestriatus* (V3)			24, 48 h	1. DNA damage	1. Induced
						22 h	1. Cell invasion	1. Inhibited ^a^
						12 h	1. Gene expression (1) Pro-apoptotic genes (2) Anti-apoptotic genes 2. Signaling protein 3. Cytokine	1. (1) p53: Upregulated ^a^ (2) Bcl-xL, BID: Downregulated ^a^ 2. STAT3, RhoC, Erk^1/2^: Inhibited ^a^ 3. IL-6: Decreased (n.s)

^a^ *p* < 0.05; ^b^ *p* < 0.01; ^c^ *p* < 0.005; ^d^ *p* < 0.001. n.r.; not reported; FaSL, Fas ligand; ALDOA, Aldolas; PKM, Pyruvate kinase; NO, Nitrite oxide; GSH, Glutathione; MMP, Mitochondrial membrane potential; ROS, Reactive oxygen species.

**Table 4:** Anticancer mechanism of scorpion bioactive protein in breast cancer cells.

Study ID	Author, Year	Target Cells	Scorpion Species	Proteins	Concentration	IC50	Duration	Outcome Measure	Results
1	D’Suze et al. (2010) [28]	SKBR3	*Tityus discrepans*	Neopladine 1,2	1, 5, 15, 30 µg/µL	n.r	1, 2, 3, 4, 5, 6 h	1. Apoptosis 2. Pro-apoptotic protein 3. Anti-apoptotic gene	1. Elevation 2. FasL: Upregulated (n.r) 3. Bcl-2: Downregulated (n.r)
18	Wang et al. (2019) [45]	MCF-7	*Leiurus quinquestriatus*	Chlorotoxin	0.05, 0.5, 5 µmol/L	-	12, 24, 48, 72 h	1. Cell proliferation 2. Cell migration and invasion 3. ERα 4. MMP2 5. VASP	1. Dose and time dependently inhibited ^a^ 2. Dose-dependently inhibited ^a^ at 24 h 3. Inhibited ^a^ 4. Inhibited ^a^ 5. Inhibited ^a^
		MDA-MB-231						1. Cell proliferation 2. Cell migration and invasion 3. ERα 4. MMP2 5. VASP	1. Dose and time dependently inhibited ^a^ 2. Dose-dependently inhibited ^a^ at 24 h 3. Inhibited ^a^ 4. Inhibited ^a^ 5. Inhibited ^a^
		T47D						1. Cell proliferation 2. Cell migration and invasion 3. ERα 4. MMP2 5. VASP	1. Dose and time dependently inhibited ^a^ 2. Dose-dependently inhibited ^a^ at 36 h 3. Inhibited ^a^ 4. Inhibited ^a^ 5. Inhibited ^a^
17	Rezaei et al. (2022) [44]	MDA-MB-231	*Hemiscorpius lepturus*	Purified Leptulipin	6.25, 12.5, 25, 50 µg/mL	30 µg/mL	24 h	1. Cell cycle (1) G0/G1 phase (2) G2/M phase 2. Gene expression (1) Pro-apoptotic genes (2) Anti-apoptotic genes 3. DNA fragmentation	1. (1) Increase ^c^ (2) Decrease ^b^ 2. (1) Upregulated: Bax ^d^, Caspase-9 ^d^ (2) Downregulated: Bcl-2 ^c^ 3. Induced at 25 µg/mL
19	Feng et al. (2008) [46]	MDA-MB-231	*Buthus martensi karsch*	BmHYA1	100U	-	20 h	1. Hyaluronan	1. Reduction ^b^
							12, 24, 48 h	1. CD44v6 expression	1. Downregulation after 48 h ^a^

^a^* p* < 0.05; ^b^ *p* < 0.01; ^c^ *p* < 0.005; ^d^ *p* < 0.001. n.r.; not reported; FaSL, Fas ligand; ERα, Estrogen-receptor α; MMP-2, Matrix metalloproteinase-2; VASP, Vasodilator stimulated phosphoprotein.

## Data Availability

Data sharing is not applicable to this article as no new data were generated or analyzed during this study. All data supporting the findings of this review are available from the cited references.

## Abbreviations

The following abbreviations are used in this manuscript:

ALDOA	Aldolas
CTX	Chlorotoxin
FaSL	Fas ligand
GSH	Glutathione
LDH	Lactate Dehydrogenase release assay
MeSH	Medical Subject Headings
MMP	Mitochondrial membrane potential
MTT	3-(4,5-Dimethylthiazol-2-yl)-2,5-Diphenyltetrazolium Bromide assay
NO	Nitrite oxide
NRU	Neutral Red Uptake assay
PKM	Pyruvate kinase
ROS	Reactive oxygen species
SERMs	Selective estrogen receptor modulators
SRB	Sulforhodamine B assay
XTT	2,3-Bis(2-Methoxy-4-Nitro-5-Sulfophenyl)-5-[(Phenylamino)Carbonyl]-2H-Tetrazolium
